# Ameliorating effect of *Erxian * decoction combined with Fructus *Schisandrae chinensis (Wu Wei Zi*) on menopausal sweating and serum hormone profiles in a rat model

**DOI:** 10.1186/s13020-016-0117-6

**Published:** 2016-11-22

**Authors:** Shi Wei Wang, Fei Hua Wu, Yan Bo Zhang, Liang Zhang, Jing Su, Hei Kei Wong, Ai Hua Liu, Ho Pan Cheung, Tzi Bun Ng, Yao Tong, Stephen Cho Wing Sze

**Affiliations:** 1 School of Chinese Medicine, LKS Faculty of Medicine, The University of Hong Kong, 10 Sassoon Road, Pokfulam, Hong Kong SAR; 2 Department of Pharmacology, China Pharmaceutical University, Nanjing, China; 3 Department of Physiology, School of Basic Medicine, Shanghai University of Traditional Chinese Medicine, Shanghai, China; 4 School of Biomedical Sciences, Faculty of Medicine, The Chinese University of Hong Kong, Shatin, N.T., Hong Kong SAR

## Abstract

**Background:**

Modified *Erxian decoction* (MEXD), i.e., *Erxian * decoction (EXD) with Fructus *Schisandrae chinensis* (*Wu Wei Zi*) added, has been used to alleviate menopausal symptoms. This study aimed to investigate the effects of MEXD on menopausal sweating and serum hormone levels in a rat model of menopause after oral administration of MEXD.

**Methods:**

Quality control of MEXD was conducted by employing a reversed-phase high performance liquid chromatography column. The three treatment groups received oral administration of MEXD in 0.5% sodium carboxylmethyl cellulose (CMC-Na) at three different doses (5.5, 11, and 22 g/kg body weight) once-daily for 6 consecutive weeks, with 10 animals per group. *Huangqijing* oral liquor (5 mL/kg) prepared from the roots of *Huang qi* (*Astragalus membranaceus*) with an antiperspirant effect was used as a positive control. The negative control group received the same volume of vehicle (0.5% CMC-Na). Ten 3-month-old Sprague–Dawley rats were used as a young group for comparison with the treatment groups (12–14 months old rats). Blood was collected from all animals after 3–6 weeks of treatment. At the end of the treatment, the uterine weight, ovarian weight, and body weight were recorded. Serum malondialdehyde contents and superoxide dismutase activities were determined by thiobarbituric acid colorimetric assays and chemoluminescence assays, respectively. Serum levels of estradiol, follicle-stimulating hormone, and luteinizing hormone were measured by radioimmunoassays. Rat foot pad assays were used to determine the antiperspirant activity of MEXD and histological examinations were conducted on plantar sweat glands.

**Results:**

Treatment with MEXD (11 g/kg) significantly inhibited sweat excretion in the menopause model rats after treatment for 3 (*P* = 0.0026) and 6 (*P* < 0.0001) weeks. The decoction markedly decreased the number of secretory cells in plantar sweat glands. In addition, MEXD (11 g/kg) significantly increased the serum estradiol levels (*P* < 0.001) and superoxide dismutase activities (*P* = 0.0405). Furthermore, MEXD (11 g/kg) markedly decreased the serum levels of follicle-stimulating hormone (*P* = 0.001), luteinizing hormone (*P* = 0.0213), and malondialdehyde (*P* = 0.01).

**Conclusion:**

Modified *Erxian decoction* significantly inhibited sweat excretion, regulated serum levels of pituitary gonadotropins and estradiol, and exhibited antioxidative effects in a rat model of menopause.

**Electronic supplementary material:**

The online version of this article (doi:10.1186/s13020-016-0117-6) contains supplementary material, which is available to authorized users.

## Background

Menopausal syndrome refers to symptoms that appear during the menopausal transition, including hot flashes, sweating, dysphoria, and insomnia. Sweating is the main menopausal vasomotor symptom, and can significantly affect the quality of life in middle-aged females [1]. More than 80% of middle-aged women suffer from vasomotor symptoms, including hot flashes and night sweats, during menopause [2, 3]. One of the underlying mechanisms involves decreased secretion of sex hormones [4] through deterioration of ovarian functions in an age-related free radical-predominant environment [5], which in turn elevates the production of the gonadotropins luteinizing hormone (LH) and follicle-stimulating hormone (FSH) [6]. This hormonal dysregulation eventually results in autonomic dysfunction, thereby disturbing the coordination of the neuroendocrine system. Hormone replacement therapy (HRT) has been adopted as the conventional treatment for menopausal syndrome to compensate for the reduction in ovarian estrogen production. However, concerns about increased risks of some chronic diseases, such as breast cancer, heart attack, and stroke, with HRT have stimulated interest in the development of safer alternative therapies [7].

Herbal medicines with estrogenic activity or enriched with phytoestrogens have frequently been employed as complementary therapies to relieve menopausal symptoms [8–15]. However, only a few studies have investigated the treatment of menopausal vasomotor symptoms using herbal medicines.

In Chinese medicine, menopausal syndrome is attributed to insufficiency of *kidney essence*, and deficiency of *kidney yin* and *yang*. At the age of about 49 years, the *kidney qi* gradually becomes insufficient, the function of *ChongRen* declines, and *Tian gui* (an important reproduction essence) begins to dry up, thereby causing the loss of reproductive capacity. Clinically, menopausal women often suffer from menstrual disorders, hot flashes, and night sweats. *Erxian* decoction (EXD) has been used in the treatment of menopausal syndrome, and consists of six Chinese medicinal herbs, namely rhizoma *Curculigo orchioides* (*Xian mao*), herba *Epimedium brevicornum* (*Yin yang huo*), radix *Morinda officinalis* (*Ba ji tian*), radix *Angelica sinensis* (*Dang gui*), cortex *Phellodendron chinense* (*Huang bo*), and rhizome *Anemarrhena asphodeloides* (*Zhi mu*) [11, 16]. In EXD, constituent herbs *C. orchioides*, *E. brevicornum*, and *M. officinalis* can warm the *kidney yang*, and was used to restore the *kidney yin*. *A. asphodeloides* goes into the central part of the body, where it removes the heat of the stomach and nourishes the *stomach Yin*. *P. chinense* goes into the lower part of the body, nourishes the *kidney yin*, and brings *pathogenic fire* back to the *kidneys*. *A. sinensis*, a medicine that nourishes *blood*, can also restore *qi*, and was used to restore both *blood* and *qi*. The night sweats during menopause arise through the deficiency of *kidney yin* and *yang*. However, a clinical study reported that EXD alone did not exert marked effects on perspiration [17]. The fruit of *Schisandrae chinensis* (*Wu wei zi*), with the pharmacologic effect of arresting discharge to decrease sweating, is commonly used to treat insomnia and as an antiperspirant clinically [17, 18]. A clinical study showed that modified EXD (MEXD) consisting of EXD with other added herbs, including the fruit of *S. chinensis*, can relieve menopausal sweating [18, 19]. However, related basic research data have not yet been reported.

This study aimed to investigate the effects of MEXD on menopausal sweating and serum hormone profiles in menopausal rats after oral administration of MEXD. We hypothesized that MEXD would alleviate the sweating symptom in menopausal females via increased expression of antioxidative enzymes and estradiol (E2), and decreased production of serum gonadotropins, LH, FSH, and malondialdehyde (MDA). As the study model, we used 12–14-month-old aged menopausal Sprague-Dawley (SD) rats with irregular estrous cycles, as well as elevated serum FSH levels and decreased serum E2 levels, in accordance with our previous publication [11]. The effects of MEXD on sweat excretion, uterine and ovarian weights, serum levels of E2, FSH, LH, and MDA, and activity of serum superoxide dismutase (SOD) were determined. The effects of MEXD on the morphology of plantar sweat glands were also examined. Functional assays of sweating after MEXD treatment were performed to validate its therapeutic efficacy.

## Methods

### Herbal materials and MEXD extract

The seven medicinal plants of MEXD, namely *C. orchioides*, *E. brevicornum*, *M. officinalis*, *A. sinensis*, *P. chinense*, *A. asphodeloides* and *S. Chinensis* (voucher no. CME140706-03, CME150906-11, CME050406-02, CME090706-12, CME100606-09, CME180806-01 and JWE030806-05 respectively) were collected from various sources and their identities were confirmed by Dr. Yanbo Zhang, School of Chinese Medicine, the University of Hong Kong based on their morphological appearances. One kilogram of each of the six component herbs of EXD, and 1 kg of *S. Chinensis* fruit (composition ratio = 12:12:10:10:9:9:12) was decocted separately with distilled water in a ratio of 10:1 (v/w) at 100 °C for one hour, and the extraction was repeated twice. The procedures followed those of our previous publication [20, 21].

### Quality control and high performance liquid chromatography (HPLC)

For evaluation of the quality consistency of MEXD extracts, nine chemical standards were utilized as markers, namely berberine, ferulic acid, icariin, jatrorrhizine, palmatine, schizandrol, schizandrol B, schizandrin A and schizandrin B. All these standard chemicals were purchased from Beijing Century Biotechnology Co., Ltd (Beijing, China). These nine selected chemical standards and three batches of MEXD extracts were accurately weighed out and extracted with 10 mL of methanol in a water bath at 60 °C for 15 min, followed by ultrasonication for 30 min. After centrifugation at 1500×*g* for 15 min, the supernatant was filtered with a 0.45 µm Millex® Syringe filter unit, and then injected in a volume of 10 μL into a HPLC. Reproducibility and linearity were estimated by repetitive injections. The external standard method, employing a series of mixed standard solutions ranging in concentration from 4.0625 to 145 μg/mL, was utilized. The column used was a reversed-phase column (XBridge® C_18_, 5 µm, 250 mm × 4.6 mm i.e., Waters, Ireland). The mobile phase consisted of acetonitrile (A) and 0.05% sodium dodecyl sulfate (SDS) in 0.1% acetic acid (B) using a gradient program of 95–70% (B) in 0–30 min, 70–70% (B) in 30–35 min, 70–50% (B) in 35–45 min, 50–45% (B) in 50–70 min, 45–35% (B) in 70–90 min, and 35–30% (B) in 90–100 min. The flow rate was 1.0 mL/min. The UV spectra from 200 to 400 nm were acquired with the photodiode array detector and chromatogram at 254 nm was extracted. Peak integration was analyzed by the software of Empower Pro, Waters®.

### Experimental animals and drug treatment

Sixty-five female SD rats aged 12–14 months (weight: 290–370 g) and 10 SD rats aged 3 months (weight: 160–190 g) were obtained from the Animal Center of Nanjing Qinglongshan. The animals were housed in an air-conditioned room at an ambient temperature of 24 °C and a relative humidity of 50–65% under automatic 12- /12-h light/dark cycles. All efforts were made to minimize suffering of the animals and to reduce the number of animals used. Animal experiment has been conducted in China Pharmaceutical University since 2007, which was in accordance with the regulations for animal care and use in Department of Health, the Government of the Hong Kong Special Administrative Region (Reference no. 07-298 and 09-92) (Additional files 1, 2). The animal studies were performed following the ARRIVE guideline (Additional file 3).

Using vaginal smear tests, 50 of the 65 rats with both irregular estrous cycles and lowered serum E2 levels were classified as menopausal rats, and selected as the experimental animals. These rats were arbitrarily divided into a negative control group and four treatment groups, with 10 rats per group. The four treatment groups received oral administration of MEXD in 0.5% sodium carboxylmethyl cellulose (CMC-Na) at three different doses (5.5, 11, and 22 g/kg body weight) or *Huangqijing* oral liquor at 5 mL/kg (Z32020370; Yangtze River Pharmaceutical Co. Ltd.), prepared from the roots of *Astragalus membranaceus* with an antiperspirant effect, as a positive control once-daily for 6 consecutive weeks. The drug ratios in MEXD (12:12:10:10:9:9:12), namely rhizoma *Curculigo orchioides* (*Xian mao*) 12 g, herba *Epimedium brevicornum* (*Yin yang huo*) 12 g, radix *Morinda officinalis* (*Ba ji tian*) 10 g, radix *Angelica sinensis* (*Dang gui*) 10 g, cortex *Phellodendron chinense* (*Huang bo*) 9 g, rhizome *Anemarrhena asphodeloides* (*Zhi mu*) 9 g, and Fructus *Schisandrae chinensis (Wu Wei Zi*) 12 g are commonly used clinically [20]. The dosage of 11 g/kg/day applied to rats corresponded to an equivalent dosage in humans [22], and thus three dosages of MEXD (5.5, 11, and 22 g/kg) were selected for this study. The negative control group received the same volume of vehicle (0.5% CMC-Na). The 10 SD rats aged 3 months were used as a young group for comparison with the aged rats.

### Rat foot pad assay for antiperspirant activity

Sweat secretion in the rats was detected by rat foot pad assays after MEXD treatment for 3–6 weeks. The method was originally developed by Helman et al. [23] and has been used in previous studies [23–25]. Briefly, the foot pads of all rats were cleaned with 70% ethanol to remove debris, and then swabbed with iodine solution (2% w/v in 95% ethanol). After evaporation of the ethanol at room temperature, the foot pads were coated with castor oil containing 50% starch for 15 min. As the sweating parts of the foot pads showed coloration by starch–iodine reactions, the colored points on the foot pads (i.e., number of sweat glands) were enumerated under a microscope and the degree of perspiration was measured.

### Measurement of serum antioxidant biochemical parameters and hormones

At 24 h after the last gavage, blood samples were collected from the femoral artery of the rats to determine the serum levels of MDA, SOD activity, E2, FSH, and LH. The collected blood was centrifuged at 1000×*g* for 10 min and the serum supernatant was stored at −20 °C until analysis. The serum MDA concentrations and SOD activities were determined by thiobarbituric acid colorimetric assays and chemoluminescence assays (Nanjing Jiancheng Bioengineering Institute, Nanjing, China), respectively. The serum levels of E2, FSH, and LH were measured by radioimmunoassays using an E2 assay kit (Cat. No. B05PZA), FSH assay kit (Cat. No. B03PZB), and LH assay kit (Cat. No. B04PZB) from Beijing North Institute of Biological Technology (China), respectively.

### Measurement of organ indices

All rats were killed by cervical dislocation after blood collection. The uteri and ovaries were excised and weighed immediately on an electronic balance. The organ indices were calculated by dividing the organ weights by the body weight.

### Histological examination

The epidermis of the foot pads was dissected into pieces, fixed in 10% formalin, and processed for paraffin sectioning. Tissue sections were stained with hematoxylin and eosin and examined under an optical microscope [26].

### Statistical analysis

All data were expressed as the mean ± standard deviation. Differences among groups were evaluated for statistical significance by one-way ANOVA followed by a Tukey multiple-comparisons test using GraphPad Prism 6.0 (GraphPad Software, USA). Values of *P* < 0.05 were considered statistically significant.

## Results

### HPLC for quality analysis

**Fig. 1:**
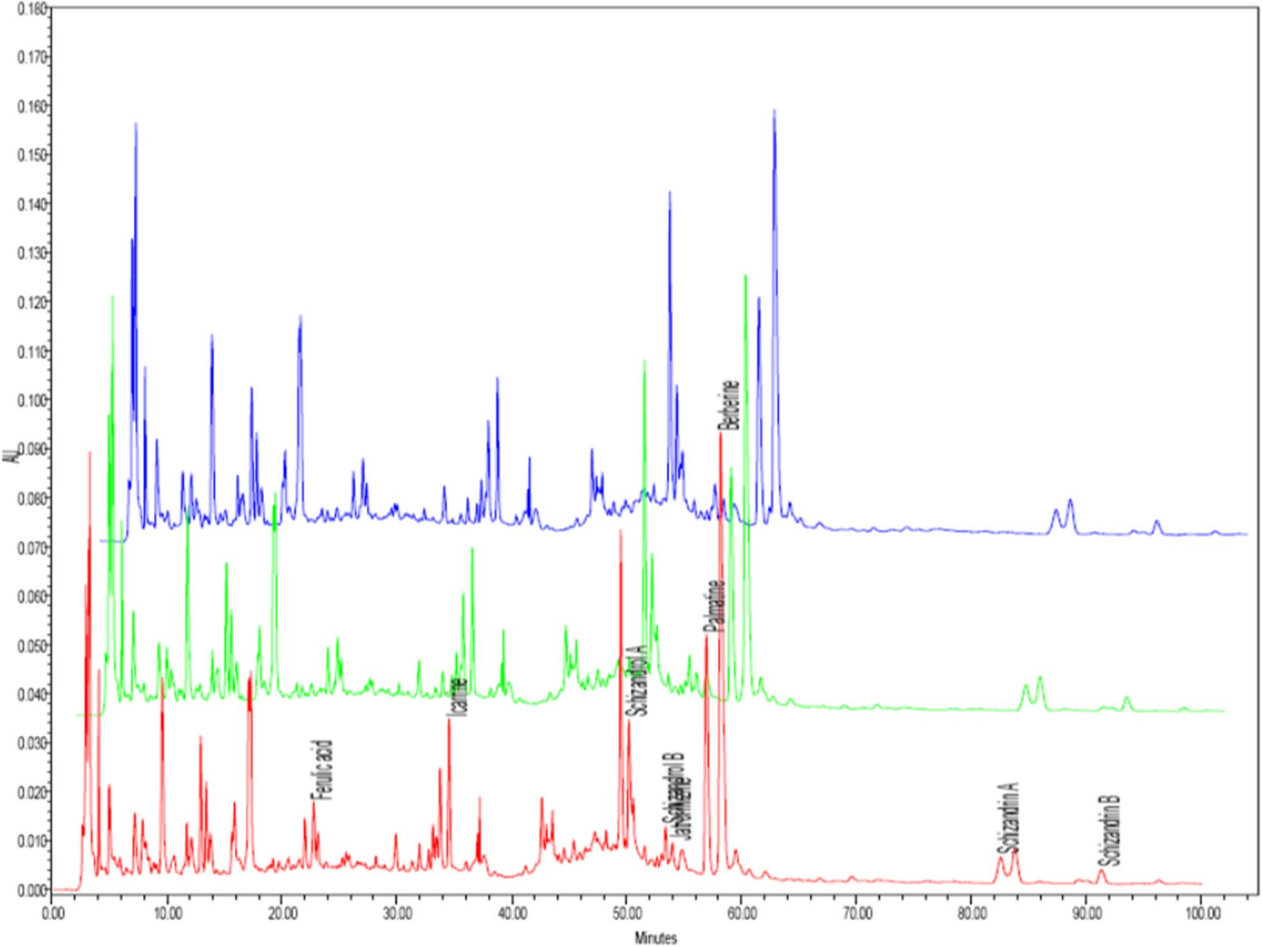
Overlaid HPLC chromatographic fingerprint of three batches of MEXD extract monitored at 254 nm. Nine standard compounds and their respective retention times are shown

The UV spectra of all eluted peaks from 200 to 400 nm in chromatograms of MEXD were measured by photodiode array detection, and the majority of the detectable peaks in the HPLC chromatograms were found at 254 nm. The chromatograms were thus extracted at the detection wavelength of 254 nm. A chromatographic fingerprint indicating the elution peaks of nine standard compounds and other common peaks is shown in Fig. 1. Three batches of MEXD preparations were examined by HPLC using the optimum running conditions. The inter assay relative standard deviation (RSD) values were less than 5% for all of the standard compounds. The results are shown in Table 1.

**Table 1:** Contents of nine compound standards in MEXD

Standards (μg/mg)	Batch 1	Batch 2	Batch 3	Mean	Standard deviation	Relative standard deviation (%)
Berberine	0.961	0.945	0.924	0.943	0.019	1.983
Ferulic acid	0.200	0.189	0.191	0.193	0.006	3.137
Icariine	0.414	0.411	0.408	0.411	0.003	0.783
Jatrorrhizine	0.054	0.054	0.053	0.054	0.000	0.078
Palmatine	0.611	0.598	0.586	0.598	0.013	2.106
Schizandrol A	0.341	0.334	0.354	0.343	0.010	2.990
Schizandrol B	0.114	0.109	0.118	0.113	0.004	3.891
Schizandrin A	0.107	0.107	0.107	0.107	0.000	0.217
Schizandrin B	0.136	0.134	0.133	0.134	0.002	1.469

### Antioxidant activity of MEXD in menopausal rats

**Fig. 2:**
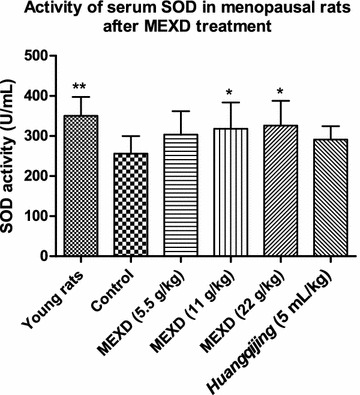
Serum levels of SOD in menopausal rats after MEXD treatment (n = 10, mean ± standard deviation). One-way ANOVA followed by multiple comparisons. *P < 0.05, **P ≤ 0.01 compared with control. Young rats vs. control: *P* = 0.0026, MEXD (22 g/kg) vs. control: *P* = 0.0474

**Fig. 3:**
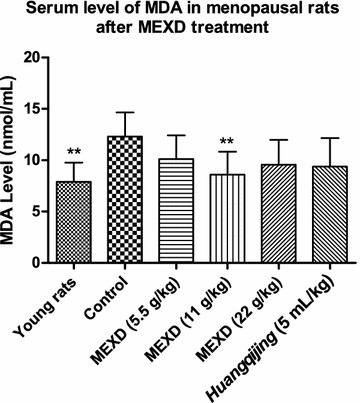
Serum levels of MDA in menopausal rats after MEXD treatment (n = 10, mean ± standard deviation). One-way ANOVA followed by multiple comparisons *P < 0.05, **P ≤ 0.01 compared with control. Young rats vs. control: *P* = 0.0013, MEXD (11 g/kg) vs. control: *P* = 0.01

The serum SOD activities were significantly lower (Fig. 2) and the serum MDA levels were significantly higher (Fig. 3) in the control group compared with the young rats. Treatment with MEXD at all dosages and *Huangqijing* was able to significantly reverse these changes.

### Regulatory effects of MEXD on serum gonadotropic and gonadal hormones in menopausal rats

**Fig. 4:**
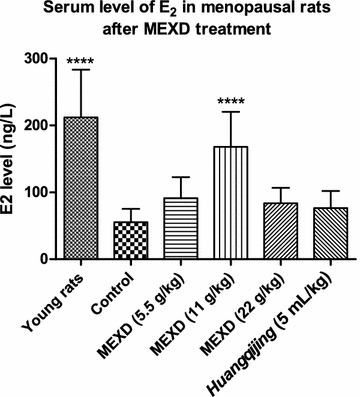
Serum levels of E2 in menopausal rats after MEXD treatment (n = 10, mean ± standard deviation). One-way ANOVA followed by multiple ***P < 0.001 compared with control. Young rats vs. control: *P* < 0.0001, MEXD (11 g/kg) vs. control: *P* < 0.0001

**Fig. 5:**
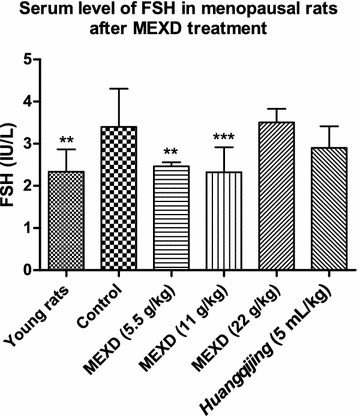
Serum levels of FSH in menopausal rats after MEXD treatment (n = 10, mean ± standard deviation).One-way ANOVA followed by multiple comparisons. **P ≤ 0.01, ***P < 0.001 compared with control. Young rats vs. control: *P* = 0.001, MEXD (5.5 g/kg) vs. control: *P* = 0.005, MEXD (11 g/kg) vs. control: *P* = 0.0008

**Fig. 6:**
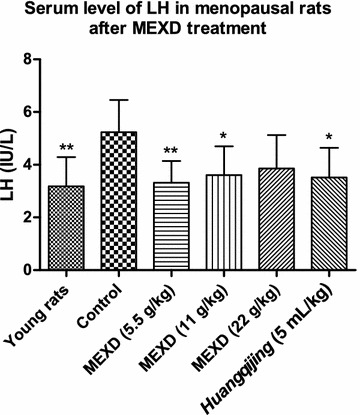
Serum levels of LH in menopausal rats after MEXD treatment (n = 10, mean ± standard deviation). One-way ANOVA followed by multiple **P ≤ 0.01 and *P < 0.05 compared with control. Young rats vs. control: *P* = 0.0017, MEXD (5.5 g/kg) vs. control: *P* = 0.004, MEXD (11 g/kg) vs. control: *P* = 0.0213*, Huangqijing* vs. control: *P* = 0.0131

Significantly reduced serum E2 levels (Fig. 4) accompanied by increased serum levels of FSH (Fig. 5) and LH (Fig. 6) were found in the menopausal rats in the control group compared with the young rats. After treatment with MEXD at 11 g/kg, the serum E2 levels increased significantly. Meanwhile, treatment with MEXD at 5.5 and 11 g/kg decreased the serum LH and FSH levels compared with the control group.

### Effects of MEXD on organ indices of the ovaries and uteri

Compared with the young rats, the organ indices of the ovaries of the menopausal rats in the control group were significantly decreased. However, the organ indices of the ovaries and uteri in all four treatment groups (MEXD and *Huangqijing*) did not differ significantly compared with those in the control group (Table 2). These findings indicated that MEXD treatment exerted no observable adverse effects on the ovaries and uteri.

**Table 2:** Organ indices of ovaries and uteruses in menopausal rats after MEXD treatment (n = 10, mean ± standard deviation)

Group	Organ index	
	Uterus (×10^−3^)	Ovary (×10^−4^)
Young	2.33 ± 0.81	3.38 ± 0.33**
Control	2.34 ± 0.58	2.30 ± 0.51
MEXD (5.5 g/kg)	1.80 ± 0.35	2.37 ± 0.67
MEXD (11 g/kg)	1.88 ± 0.32	2.21 ± 0.25
MEXD (22 g/kg)	2.23 ± 0.63	2.54 ± 0.84
*Huangqijing* (5 mL/kg)	2.00 ± 0.38	2.27 ± 0.47

** *P* < 0.001 (one way ANOVA, n = 10) compared with control

### Antiperspirant activity of MEXD on the foot pads of menopausal rats

**Fig. 7:**
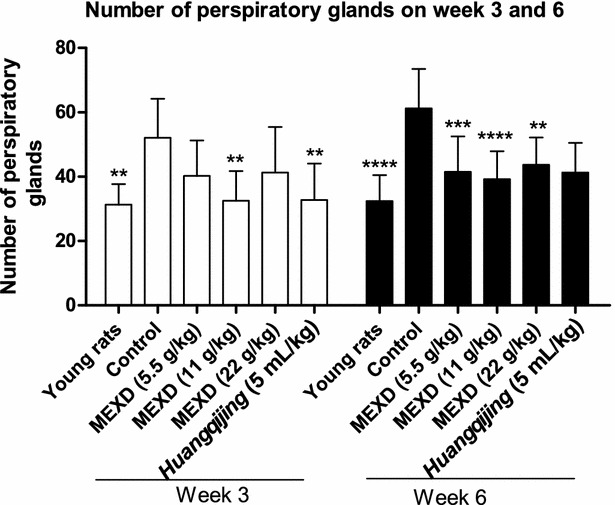
Antiperspirant effect of MEXD on rats foot pads (n = 10, mean ± standard deviation) one-way ANOVA followed by multiple. *P < 0.05, **P ≤ 0.01 and ***P < 0.001 compared with control. *Week 3* young rats vs. control: *P* = 0.0012, MEXD (11 g/kg) vs. control: *P* = 0.0026*, Huangqijing* vs. control: *P* = 0.0032. *Week 6* young rats vs. control: *P* < 0.0001, MEXD (5.5 g/kg) vs. control: *P* = 0.0005, MEXD (11 g/kg) vs. control: *P* < 0.0001, MEXD (5.5 g/kg) vs. control: *P* = 0.0034

Sweat secretion of rat foot pads was determined by the iodine–starch reaction in all groups and the results are shown in Fig. 7. The number of active sweat glands in the menopausal rats in the control group was significantly higher than that in the young rats (*P* = 0.0012). At the dosage of 11 g/kg, MEXD significantly decreased the number of sweat glands in the menopausal rats after 3 weeks of treatment. All dosages of MEXD were able to inhibit sweat secretion, especially the dose of 11 g/kg, which significantly decreased the number of sweat glands (*P* < 0.001) in the menopausal rats after 6 weeks of treatment.

**Fig. 8:**
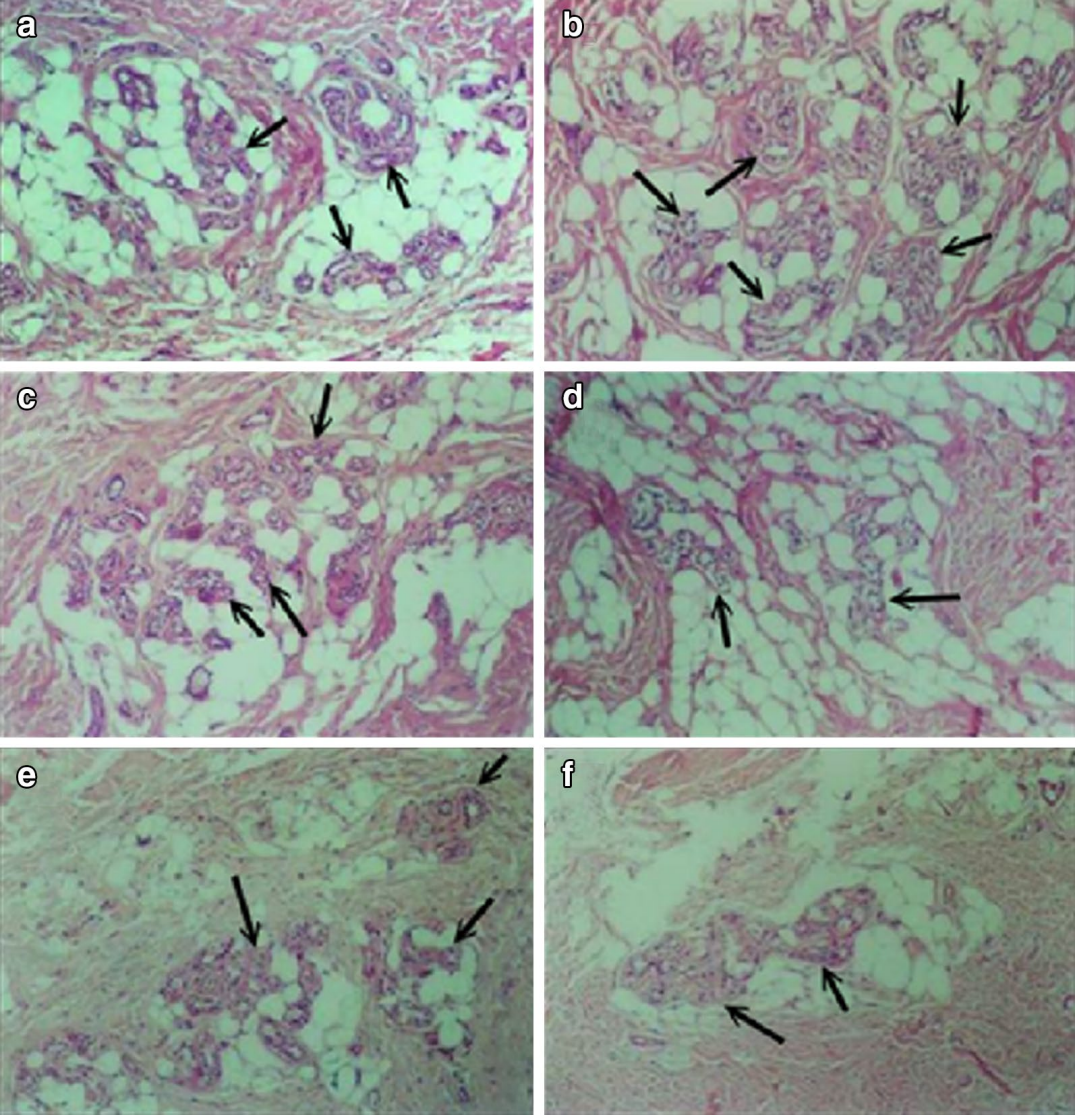
Secretory portions of rat plantar sweat glands in foot pads (indicated by *solid black arrows*). **a** Young rats; **b** control; **c**–**e** MEXD (5.5, 11 and 22 g/kg) treated menopausal rats; **f** *Huangqijing* (5 mL/kg) treated menopausal rats. (H&E stain × 100)

In the plantar sweat glands of the young rats, the secretory portions lay in clusters among the fat cells of the hypodermis. Irregular connective tissue of the dermis and numerous blood vessels were in the vicinity of the secretory portions (Fig. 8a). In the plantar sweat glands of the menopausal rats in the control group, the number of secretory cells had increased (Fig. 8b). Both the number of secretory cells and the number of vacuoles in the cells were significantly lower in the plantar sweat glands of the menopausal rats after treatment with all dosages of MEXD and *Huangqijing* (Fig. 8c–f).

## Discussion

In this study, the anti-menopausal sweating effects of MEXD were evaluated using a 12–14-month-old aged menopausal SD rat model with both irregular estrous cycles and lowered serum E2 levels. All animal models have some limitations, and in this case, the age and physiology of the rats cannot completely mimic human female menopause. The duration of the estrous cycle in SD rats is 3–4 days [27], and the lifespan of the rats is only 2.5–3.5 years [28], while the human menstrual cycle occurs at approximately monthly intervals. During menopause, the ovarian function declines, but the ovaries still synthesize some steroid hormones such as androstenedione, testosterone, and E2 [29]. Thus, the use of ovariectomized female SD rats as an animal model of menopause cannot mimic the physiological states of menopause and ovarian aging in humans. A previous study demonstrated that SD rats have a fertility duration of 3–9 months, indicating that rats aged 12 months and older would correspond to the perimenopause phase in humans [28]. Our unpublished data showed that 12-month-old SD rats undergoing natural aging demonstrated markedly reduced circulating E2 levels compared with 3-month-old rats, indicating that these rats may mimic the human female entering the menopause transition phase. Therefore, our animal model employing 12–14-month-old female SD rats undergoing natural aging is reasonable. In this study, the estrous phase was determined by vaginal smears, and rats with irregular estrous cycles were regarded as menopausal rats.

To exclude variations in bioactivity caused by inconsistency of herbal extract preparations, HPLC was used to evaluate the quality consistency of the MEXD extract prior to the animal experiments. Nine standard compounds, namely berberine, ferulic acid, icariin, jatrorrhizine, palmatine, schizandrol A, schizandrol B, schizandrin A, and schizandrin B, were selected as markers for evaluating the quality of MEXD (Fig. 1).

Five of these standard chemicals (jatorrhizine, berberine, palmatine, ferulic acid, and icariin) are well-known compounds in EXD according to the Pharmacopoeia of the People’s Republic of China [30] and our previous publication [11]. All of these chemicals possess antioxidant activities. Moreover, three of them (ferulic acid, icariin, and berberine) exhibit estrogenic activity. Therefore, these constituents used for quality control are important for the biological activity of the extract. Meanwhile, the markers for *S. chinensis* were not only the major components, but also possessed related pharmaceutical activities [31], including estrogenic and antioxidant activities. Besides, polysaccharides, free amino acids, lignans including schizandrol A, schizandrol B, schizandrin A, and schizandrin B were the major components in *S. chinensis*, and have been commonly used for quality control of *S. chinensis* [32–34]. Based on modern pharmacological studies, schizandrol A and schizandrin B possess antioxidant properties [35]. Schisantherin A manifests estrogenic effects [36]. The interassay RSD values were less than 5% for all nine standard compounds, indicating good quality of the MEXD extract and consistency between different batches of the MEXD extract (Table 1).

Aging is associated with higher levels of oxidative biomolecules reacting with free radicals [36–38]. Menopause or ovariectomy (estrogen deficiency) can induce hot flashes and oxidative stress [38]. In addition, reactive oxygen species attack the heme group of aromatase to inhibit E2 biosynthesis [39]. Previous studies demonstrated that pulsatile release of FSH and LH is related to the mechanism underlying the initiation of hot flashes [40, 41]. Commonly, HRT effectively decreases oxidative stress and relieves hot flashes [42], indicating that estrogen deficiency may increase oxidative stress and hot flashes physiologically. Besides, other studies indicated that increased oxidative stress and MDA levels and decreased antioxidant enzyme SOD levels accompany hot flashes in menopausal women, and these symptoms were reversed after receiving HRT or undertaking yoga exercise [43–45]. The increased production of oxygen free radicals concomitant with aging accelerates the depletion of SOD and hinders SOD synthesis, leading to decreased SOD activity. In this study, the serum SOD activities of menopausal rats in the control group were significantly lower compared with those in the young rats (Fig. 2). The increased MDA contents indicated that lipid peroxidation became more active in menopausal rats, and the antioxidant abilities were declining. Our results revealed that MEXD at the dose of 11 g/kg effectively reduced serum MDA levels and promoted SOD activities (Figs. 2, 3). These findings demonstrate that MEXD treatment can improve the antioxidant ability in aged female rats and potentially slow down the aging process.

The antioxidant activity of MEXD indicates its role in preventing reproductive aging. Along with reproductive aging, the activities of antioxidant enzymes and steroidogenic enzymes including aromatase become lower, suggesting the association between an age-related environment predominated by free radicals and a decrease in estrogen production through the effect on aromatase (Cyp19) activity in the human ovary [5]. It is known that E2 synthesis is mainly mediated by aromatase in ovarian granulosa cells [46]. Our previous publication [11] demonstrated that one of the possible mechanisms underlying the EXD-mediated relief of menopausal syndrome involves increases in endocrine function and serum E2 levels through activation of ovarian aromatase. Moreover, recent findings suggest that the depletion of estrogen in the postmenopausal period can cause oxidative stress in addition to the known symptoms [47], given that E2 itself possesses antioxidative properties. Oxidative stress stimulates hydroperoxide-dependent hydroxylation of E2 to catecholestrogen metabolites, which in turn can undergo reactive oxygen species-producing redox cycling, thus setting up a self-generating toxic cascade offsetting the antioxidant effects generated by the parent E2 [48]. In this study, treatment of menopausal female rats with MEXD promoted the serum activities of the antioxidative enzyme SOD (Fig. 2), thus possibly preventing the damaging effect of free radicals on the aging ovaries.

Apart from the oxidative stress theory of reproductive aging, it is known that as the number of immature follicles declines with age, the negative feedback control of E2 on FSH secretion is relaxed, and therefore the basal circulating level of FSH increases. FSH orchestrates the termination of folliculogenesis in ovaries, and high levels of FSH promote inappropriate maturation of the granulosa cells of the residual prenatal follicles. This asynchronous maturation of the germinal and somatic components results in follicular atresia, leading to a decrease in E2 production [49]. Among the MEXD-treated groups, MEXD at the dosage of 11 g/kg restored the serum E2 levels (Fig. 4) and significantly reversed the increased FSH (Fig. 5) and LH (Fig. 6) levels compared with the control group. These results demonstrated that MEXD can improve the estrogen-deficient state in aged female rats and prevent reproductive aging. Moreover, the weights of the ovaries and uteri as well as their organ indices did not change after MEXD treatment, indicating that MEXD does not exert any observable adverse effects on the rat ovaries and uteri.

The regulatory effects of MEXD on menopausal hormone profiles provide insights into interventions for vasomotor symptoms during menopause. According to the Dynamic Hypothesis, the most widely-accepted current theory [4, 50], hot flashes and sweating result from the dynamic reduction or sudden deprivation of sex hormones rather than their absolute plasma concentrations. Application of MEXD may reverse the estrogen decline during menopause, and thus prevent hot flashes and sweating. To elucidate the effects of MEXD on menopausal sweating, *Huangqijing* oral liquor, an oral preparation composed of *Radix Astragali* extract, with the functions of replenishing *Qi*, nourishing *blood*, and reinforcing *vital Qi* to stop sweating according to Traditional Chinese Medicine theory [51], was selected as the positive control. Our results revealed that sweating in the soles of menopausal rats in the control group increased significantly, while MEXD at the dosage of 11 g/kg significantly reduced such sweating after both 3–6 weeks of treatment (Fig. 7). The results of the histological study also demonstrated an increase in the number of sweat glands in the soles of the menopausal rats (Fig. 8b). On the contrary, the MEXD-treated groups at all dosages (5.5, 11 and 22 g/kg) exhibited conspicuous decreases in the number of secretory cells (Fig. 8c–e). Therefore, the potential efficacy of MEXD against menopausal perspiration involves increases in endocrine function and serum E2 levels through the antioxidant activity pathways.

In Chinese medicine, through insufficiency of *yin*, *yang* cannot be preserved and the asthenia *yang* cannot guard *yin*, and thus *yin* is excreted in the form of sweat. Therefore, clinically, for restoring the *kidney yin* and *yang*, clinicians usually add *S. chinensis*. In Chinese medicine, the flavor of *S. chinensis* is sour, which can restrain *body essence* and nourish *yin*. *A. sinensis* together with *S. chinensis* can restore both *liver blood* and *kidney essence*, which is in accord with the theory of Chinese medicine that the liver and kidney share the same source. Taken together, the whole formula can nourish *kidney yin* by warming the *kidney yang*, and nourish *yin* to preserve asthenia *yang*, and when yin and *yang* reach a balance, menopausal sweating will cease. Chinese medicine mentions that *Tian gui* may refer to steroid hormones, including E2, which play a vital role in the reproductive system and the process of female menopause. In Chinese medicine, kidney-nourishing herb medicines can restore *Tian gui*, which mimics HRT in supplementing E2. This study also indicated that MEXD was able to promote E2 biosynthesis (Fig. 4). Oral HRT was reported to be highly effective in alleviating hot flushes and night sweats [52]. The results of the present study using a menopausal rat model not only provide evidence to support the traditional Chinese medicine theory, but also shed new light on the effects of drugs in alleviating menopausal sweating and menopausal vasomotor symptoms. Interestingly, our study showed that the pharmacological activities of a Chinese medicine formula are different from those a pure chemical drug, as they did not show a dose-dependent response. This finding also indicates that the complexity of Chinese medicines may be attributed to the interactions among multiple compounds within herbal medicines, and that their interactions in the body should be mediated in the manner of a network of intermolecular interactions. Further studies should investigate the unique pharmacological activities of this herbal formula.

In essence, sweating falls within the scope of automatic temperature regulation. Previous studies suggested that hot flashes, consisting of sweating and peripheral vasodilation, are triggered by small elevations in the core body temperature (CBT) acting within a reduced thermoneutral zone in symptomatic postmenopausal women [53]. The narrowing of the zone may be caused by elevated central noradrenergic activation and is probably precipitated by changes in estrogen [54]. Estrogen withdrawal increases the sensitivity of thermoregulatory neural pathways and modifies the activation of heat loss mechanisms. It was also reported that postmenopausal women with symptomatic hot flashes have a significantly lower rectal temperature for the sweating threshold, and their sweating rate is elevated, compared with their premenopausal counterparts [53]. In other words, sweating or hot flashes can reflect thermoregulatory dysfunction in menopausal females. If the effects of MEXD can be identified by dynamic CBT monitoring, we speculate that the alleviation of menopausal sweating by MEXD may be induced by restoration of the narrowed thermoneutral zone. Further studies on the mechanism through which MEXD ameliorates menopausal perspiration are in progress.

## Conclusion

Modified *Erxian decoction* significantly inhibited sweat excretion, regulated sex hormones, and exhibited antioxidative effects in menopausal model rats.

## Additional files

**Additional file 1.** Licence to Conduct Experiments, Department of Health, the Government of the Hong Kong Special Administrative Region (Reference no. 07-298).

**Additional file 2.** Licence to Conduct Experiments, Department of Health, the Government of the Hong Kong Special Administrative Region (Reference no. 09-92).

**Additional file 3.** The animal studies were performed following the ARRIVE guideline.

## Abbreviations

EXD: *Erxian* decoction
MEXD: modified *Erxian* decoction
HRT: hormone replacement therapy
LH: luteinizing hormone
FSH: follicle-stimulating hormone
SD: Sprague–Dawley
E2: estradiol
MDA: malondialdehyde
SOD: superoxide dismutase
CMC-Na: sodium carboxylmethyl cellulose
TBA: thiobarbituric acid
R.S.D.: inter-assay relative standard deviation
