# Supplementary material for: Ameliorating effect of Erxian decoction combined with Fructus Schisandrae chinensis (Wu Wei Zi) on menopausal sweating and serum hormone profiles in a rat model
Source: Chin Med. 2016 Nov 22;11:47. doi: 10.1186/s13020-016-0117-6 (PMC5118901; doi:10.1186/s13020-016-0117-6)
Supplement: Supplementary file 1 — Additional file 1. Licence to Conduct Experiments, Department of Health, the Government of the Hong Kong Special Administrative Region (Reference no. 07-298). [file 13020_2016_117_MOESM1_ESM.pdf]

香港特別行政區政府
衞生署
醫護機構註冊辦事處

香港灣仔皇后大道東 213 號
胡忠大廈 18 樓

THE GOVERNMENT OF THE HONG KONG SPECIAL ADMINISTRATIVE REGION
DEPARTMENT OF HEALTH
OFFICE FOR REGISTRATION OF HEALTHCARE INSTITUTIONS

18/F., WU CHUNG HOUSE,
213 QUEEN'S ROAD EAST, WAN CHAI,
HONG KONG

本署檔號 OUR REF.: (07-298) in DH/ORHI/8/2/3 pt.14
來函檔號 YOUR REF.:
電　話 TEL.: 2961 8507
傳　真 FAX: 2126 7515

26 July 2007

SZE Cho-wing
School of Chinese Medicine,
The University of Hong Kong

Dear Sir/Madam,

Animals (Control of Experiments) Ordinance
Chapter 340

I refer to your application/letter dated 16.7.2007 and forward herewith the following licence(s) issued/~~duly renewed~~ under the above Ordinance :-

Form 2 : Licence to Conduct Experiments

Your attention is drawn to regulations 4 and 5 of the Animals (Control of Experiments) Regulations, copies of these regulations together with copies of Forms 6 and 7 are enclosed for your convenience. Failure to comply with either regulation 4 or regulation 5 is an offence, each offence punishable by a fine of HK$500 and to imprisonment for 3 months. Conviction of an offence against either regulation 4 or regulation 5 or failure to comply with either regulation may result in your licence being cancelled.

Please also be reminded that if you wish to continue your experiments after the specified periods as stated on the above licence / endorsements / teaching permit, you should renew them at least one-month before the end-dates. On the other hand, if you have completed or stopped your experiments before the specified periods, you should inform this Office immediately.

Yours sincerely,

(Dr KH CHAN)
for Director of Health

* *Remarks:* -

A "Code of Practice - Care and Use of Animals for Experimental Purposes" was prepared by the Agriculture, Fisheries and Conservation Department (AFCD) on the advice of the Animal Welfare Advisory Group.

Please visit the AFCD website at <http://www.afcd.gov.hk/quarantine/am/dog/code.htm> for details of the Code of Practice.

竭誠服務　顧客為本　素質為先
We are committed to providing client-oriented service

DH 1110ET

Form 2

Licence to Conduct Experiments

Name : SZE Cho-wing [Ref No.: (07-298) in DH/ORHI/8/2/3 pt.14]

Address : School of Chinese Medicine
The University of Hong Kong

By virtue of section 7 of the Animals (Control of Experiments) Ordinance, Chapter 340, the above-named is hereby licensed to conduct the type of experiment(s), at the place(s) and upon the conditions, hereinafter mentioned.

Type of experiment(s)

SD rats and BALB/c mice will be used in this experiment. Chinese medicine decoction (EXD) will be given to animals through oral administration for a period of 1 to 2 months. After a 60 days treatment period, the animal will be immediately sacrificed by an intraperitoneal overdose of ketamine/xylazine. Organs such as spleen, kidney and liver will be harvested for further studies on RT-PCR, immunoblotting and immunohistochemistry analysis.

Place(s) where experiment(s) may be conducted

1/F., School of Chinese Medicine, The University of Hong Kong, 10 Sassoon Road, Hong Kong.

Conditions

(1) Such experiment(s) may only be conducted for the following purposes :-

To study the molecular actions of a modern Chinese medicine decoction (EXD) in *in vitro* immunomodulation using animal model.

(2) This licence is valid from 26 July 2007 to 25 July 2009

Dated 26 July 2007

DEPARTMENT OF HEALTH 衞生署 HONG KONG

(Dr. Monica WONG)
for Director of Health
Licensing Authority
