# Supplementary material for: Ameliorating effect of Erxian decoction combined with Fructus Schisandrae chinensis (Wu Wei Zi) on menopausal sweating and serum hormone profiles in a rat model
Source: Chin Med. 2016 Nov 22;11:47. doi: 10.1186/s13020-016-0117-6 (PMC5118901; doi:10.1186/s13020-016-0117-6)
Supplement: Supplementary file 2 — Additional file 2. Licence to Conduct Experiments, Department of Health, the Government of the Hong Kong Special Administrative Region (Reference no. 09-92). [file 13020_2016_117_MOESM2_ESM.pdf]

Form 2

Licence to Conduct Experiments

Name : SZE Cho Wing [Ref No.: (09-92) in DH/HA&P/8/2/3 Pt.10]

Address : School of Chinese Medicine, The University of Hong Kong

By virtue of section 7 of the Animals (Control of Experiments) Ordinance, Chapter 340, the above-named is hereby licensed to conduct the type of experiment(s), at the place(s) and upon the conditions, hereinafter mentioned.

Type of experiment(s)

Rats will be used in the experiment. Chinese medicine decoction, its fraction, compound, protein and peptide will be given to the animals via oral administration or intraperitoneal injection for a period of 1 to 2 months. After treatment, the animals will be sacrificed by overdose of anaesthetic. Organs (such as spleen, liver and kidney) will be harvested for further analyses.

Place(s) where experiment(s) may be conducted

1/F, 10 Sassoon Road, Pokfulam, School of Chinese Medicine, The University of Hong Kong

---

Conditions

1. Such experiment(s) may only be conducted for the following purposes-
   To study the molecular actions of a Chinese medicine in animal model.

2. This licence is valid from 25 March 2009 to 24 March 2011

Dated 25 March 2009

DEPARTMENT OF HEALTH 衞生署 HONG KONG

Licensing Authority
